# Identifying COVID19 from Chest CT Images: A Deep Convolutional Neural Networks Based Approach

**DOI:** 10.1155/2020/8843664

**Published:** 2020-08-11

**Authors:** Arnab Kumar Mishra, Sujit Kumar Das, Pinki Roy, Sivaji Bandyopadhyay

**Affiliations:** Department of CSE, National Institute of Technology, Silchar, India

## Abstract

Coronavirus Disease (COVID19) is a fast-spreading infectious disease that is currently causing a healthcare crisis around the world. Due to the current limitations of the reverse transcription-polymerase chain reaction (RT-PCR) based tests for detecting COVID19, recently radiology imaging based ideas have been proposed by various works. In this work, various Deep CNN based approaches are explored for detecting the presence of COVID19 from chest CT images. A decision fusion based approach is also proposed, which combines predictions from multiple individual models, to produce a final prediction. Experimental results show that the proposed decision fusion based approach is able to achieve above 86% results across all the performance metrics under consideration, with average AUROC and F1-Score being 0.883 and 0.867, respectively. The experimental observations suggest the potential applicability of such Deep CNN based approach in real diagnostic scenarios, which could be of very high utility in terms of achieving fast testing for COVID19.

## 1. Introduction

The ongoing pandemic of Coronavirus Disease (COVID19) is causing a serious global crisis at the moment. With over 12 million COVID19 positive cases around the world, causing over 550,000 deaths already (according to World Health Organization statistics: https://www.who.int/emergencies/diseases/novel-coronavirus-2019), this pandemic poses the biggest healthcare threat towards humanity as of now. At present, the tests for detecting the presence of COVID19 are performed based on *reverse transcription-polymerase chain reaction (RT-PCR),* which usually takes 4–6 hours to generate results. Apart from this, availability of testing kits poses another serious problem in terms of efficient detection of the disease. To deal with these issues, radiology imaging based approaches have been proposed by multiple works recently [1–4], showing great promise for application of Artificial Intelligence (AI) and deep learning (DL) based approaches for efficient detection of the disease from chest CT images. Motivated by the need for extensive evaluation of such AI based approaches, in this work, Deep CNN based strategies are explored and studied experimentally to assess the usefulness of the approaches in the present crisis.

The field of medical imaging has seen revolutionary changes in recent past, due to the advancements in the field of DL and Computer Vision (CV), for many diseases, like Cancer, Pneumonia, ARDS, MERS, SARS, and so forth. During the current COVID19 pandemic, it has become even more important for such DL based approaches to be used in real time. Successful application of such DL based approaches can potentially be of very high utility, especially with respect to fast testing and detection of the disease.

The application of 3D DL based framework was studied by Li et al. [1], where the proposed approach was trained using a CT image dataset that consisted of COVID19, Commonly Acquired Pneumonia (CAP), and Non-Pneumonia CT scans. The goal was to differentiate between COVID19, CAP, and Non-Pneumonia cases. Wang et al. [2] proposed the use of Deep CNN based approach on CT images for differentiating between COVID19 and typical viral pneumonia cases, achieving a 73% percent accuracy. A DL based approach with local attention based mechanism was studied by Xu et al. [3] to distinguish between COVID19, Influenza-A Viral Pneumonia, and healthy CT scans. However, the datasets used in these studies have not been released publicly, due to privacy related concerns. Recently, Zhao et al. [4] have released a CT scan dataset publicly, consisting of images collected from various research articles published in this domain. The authors also tested the application of DenseNet based architectures for distinguishing between COVID19 positive and negative cases, achieving an accuracy of 84.7%, with F1-Score and AUROC values being 0.853 and 0.824, respectively. A joint classification and segmentation system (JCS) was proposed by Wu et al. [5], where on CT scan images CNNs based models have been used to distinguish between positive and negative cases of the disease and to discover the fine-grained lesion area from which the images segmentation has been done. In another similar work, Amyar et al. [6] have proposed a deep learning model using a combination of COVID19, normal, lung cancer, and other types of pathological chest CT images for classification and segmentation of COVID19. Using a single encoder and two decoders for reconstruction and segmentation, a multilayer perceptron for classification, they have achieved significant Dice Coefficient and ROC value. With limited resources, Light CNNs based models perform significantly in medical image assessments. Polsinelli et al. [7] have proposed a SqueezeNet based model to distinguish COVID19 CT images from other images and reported 85% sensitivity and 0.8333 F1-Score. Apart from CT images, it is equally important to explore other image modalities and how they are significant in COVID19 detection. Born et al. [8] have used a lung ultrasound (POCUS) dataset for detection of COVID19. Their deep learning model (POCOVID-Net) copied convolution part of VGG16 and for the dense part one hidden layer with 64 neurons and subsequent ReLU activation, dropout, and batch normalization layer was followed by an output layer with softmax activation pretrained on ImageNet to extract features from the images. They have reported a sensitivity of 0.96 and F1-Score of 0.92 using 5-fold cross validation. In another similar work, Tsiknakis et al. [9] have proposed COVID19 identification using Transfer Learning on chest X-ray image data. Parameter settings sometimes play an important role in getting significant results by the deep learning models. Talha Anwar et al. [10] have used cyclic learning rate, reduce on plateau, and constant learning rate to train EfficientNet-B4 [11] to distinguish between COVID19 and normal cases in chest CT images and concluded that reduce on plateau learning rate selection strategy outperformed the other two by giving 0.9 F1-Score on model evaluation. DenseNet201 [12] has already proved its capability in object identification. To evaluate its predictive power in COVID19 identification using CT images, Jaiswal et al. [13] have used pretrained DenseNet201 on ImageNet for features extraction and modified dense layers to get the final output. In another work, 3D CT images have been utilized in diagnosis of COVID19. Zheng et al. [14] have used pretrained UNet [15] for segmenting 3D lung images and then segmentation parts are applied for prediction of infected regions using deep learning techniques. In this current work also, the dataset released by [4] is used (collected as of April 5, 2020) for performing experimental evaluation of various popular DL approaches like VGG16, ResNet50, InceptionV3, and DenseNet architectures. The experimental results suggest that the proposed decision fusion based approach, combining the decisions of all of these models, can achieve impressive efficiency in detecting the disease.

The rest of the paper is organized as follows: in Section 2, the dataset and methodology used are described, followed by the experimental results and discussion in Section 3. Finally the paper is concluded by pointing out the key findings in the paper, in addition to some future work directions to potentially improve the performance of the proposed approach.

## 2. Materials and Methods

### 2.1. Materials

**Figure 1:**
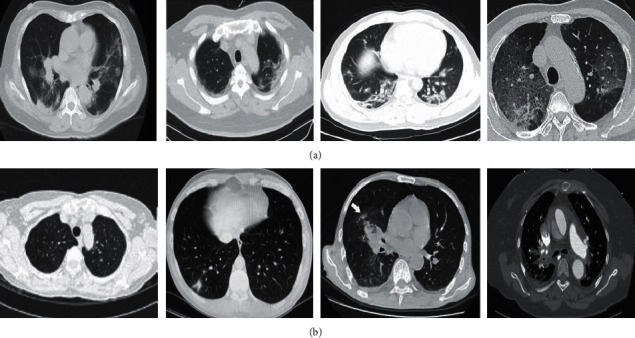
(a) Examples of positive COVID19 CT scan images. (b) Examples of non-COVID19 CT scan images.

The COVID-CT dataset [4] contains 360 positive COVID19 cases and 397 negative Chest Computed Tomography images. The positive images were collected from medRxiv and bioRxiv. These CT images are in different sizes corresponding to height ((maximum = 1853, average = 491, and minimum = 153) and width (maximum = 1485, average = 383, and minimum = 124). Some sample COVID19 positive and negative CT images are shown in Figures 1(a) and 1(b), respectively.

To prepare our final dataset for experiments, all the images have been converted into Portable Network Graphics (.png) format to keep homogeneous characteristics. Further, both positive and negative class images were resized to 224 ✕ 224 ✕ 3.

### 2.2. Methods

Recent advancements in the field of DL, especially in the medical imaging domain, indicate the potential usage of various Deep CNN architectures. Firstly, in this work, such individual baseline models are extensively evaluated. These baseline models include VGG16, InceptionV3, ResNet50, DenseNet121, and DenseNet201. In this work, all of these baseline models' convolution parts are kept exactly the same as the standard models, as proposed originally for the ImageNet challenge; however, the fully connected parts of the models are fixed as 3 fully connected layers (4096, 4096, and 1000), each with ReLU activation and finally a single-node prediction layer with Sigmoid activation function. Apart from these baseline models, a decision fusion based approach is also considered in this work.

**Figure 2:**
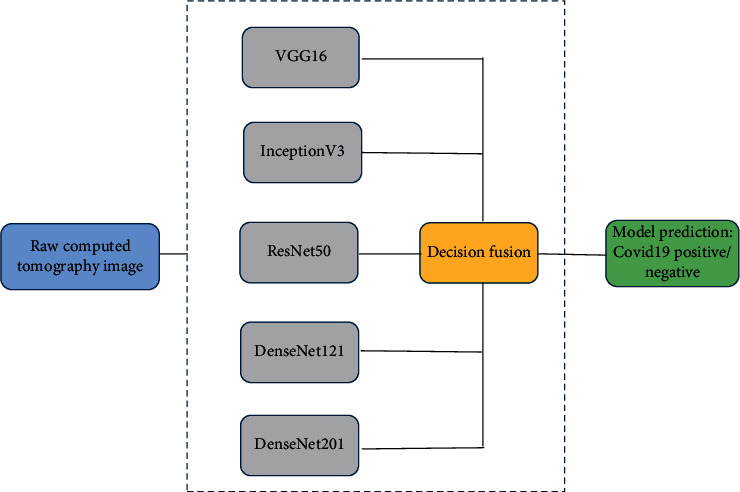
Deep CNN based decision fusion model.

**Figure 3:**
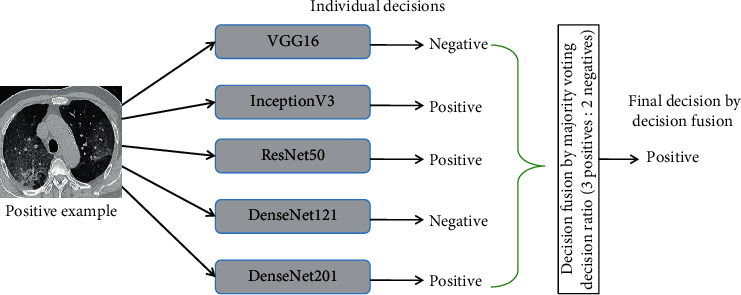
Illustration of decision fusion.

The main idea of this decision fusion approach is that the mistakes of individual models may be dealt with by combining the individual predictions via majority voting approach, which can potentially improve the overall efficiency of the baseline models. The pictorial representation of the proposed model is shown in Figure 2 and the decision fusion approach with an example is illustrated in Figure 3. In the following subsections, these individual models are briefly discussed.

#### 2.2.1. VGG16 Architecture

VGG Net, proposed by Simonyan and Zisserman [16] from the Visual Geometry Group at University of Oxford, is by far one of the most popular Deep CNN architectures, which secured the 1st and 2nd positions in the ILSVRC 2014 object localization and classification tasks. In this architecture, the main idea was that increasing the depth of the CNN architectures and replacing large kernels by multiple smaller kernels were potentially more accurate in carrying out Computer Vision tasks. VGG Net variants are still used quite extensively for many Computer Vision tasks for extracting deep image features, for further processing, especially in the medical imaging field.

#### 2.2.2. InceptionV3 Architecture

In the InceptionV3 architectures, the main idea is to deal with the problem of extreme variability in the location of the salient parts in the images under consideration by letting the network contain multiple different types of kernels in the same level, which essentially “widens” the network. This idea of multiple kernels at the same level is realized by what are called the Inception modules. With this key idea, the first InceptionV1 (GoogLeNet) [17] was proposed. Later on, in [18], InceptionV2 and InceptionV3 architectures were proposed, which improved on the InceptionV1 architecture by addressing key issues regarding representational bottleneck and auxiliary classifiers, by adding kernel factorization, and by adding batch normalization to auxiliary classifiers. This InceptionV3 architecture was the 1st runner up in the ILSVRC 2015 image classification task.

#### 2.2.3. ResNet50 Architecture

The key idea in ResNet architectures, introduced in He et al.'s work [19], is that stacking up of convolutional and pooling layers one on top of another, can cause the network performance to degrade, due to the problem of vanishing gradient, so, to deal with this, identity shortcut connections can be used, which can basically skip one or more layers. These sets of layers that contain identity connections are called a residual block. The idea of adding skip connections essentially gets rid of the high training error, which is typically observed in an otherwise deep architecture. ResNet50 is one of the variants of the ResNet architecture that contains 50 layers.

#### 2.2.4. DenseNet Architecture

The DenseNet architecture, proposed by Huang et al. [12], improves on ResNet architecture by incorporating dense connections, which essentially connect each layer to every other layer. This kind of densely connected architectures ensure that each layer gets the feature maps from each preceding layer and passes on its own feature map to each subsequent layer. Another important advantage of such an architecture is the ability to reuse features, while maintaining a low number of parameters in total. There are multiple variants of the DenseNet architecture which are used widely, among which DenseNet121 and DenseNet201 architectures are utilized in this work.

## 3. Experimental Results and Discussion

In order to perform experimental evaluation of the models under consideration, various performance metrics like Accuracy, AUC value of the ROC curve, F1-Score, Sensitivity, Specificity, Precision, and Recall are used in this work. These evaluation metrics are particularly useful while evaluating a medical screening system, which is why they are chosen for the task of COVID19 prediction also. The definitions for each of these performance metrics are given below.

(1) $$\begin{array}{c} \mathrm{Accuracy}\;=\frac{\left(\mathrm{TP}\;+\mathrm{FN}\right)}{\left(\mathrm{TP}+\;\mathrm{TN}\;+\;\mathrm{FP}\;+\;\mathrm{FN}\right)},\\ \mathrm{Sensitivity}\;=\;\frac{\mathrm{TP}}{\left(\mathrm{TP}\;+\;\mathrm{FN}\right)},\\ \mathrm{Specificity}\;=\;\frac{\mathrm{TN}}{\left(\mathrm{TN}\;+\;\mathrm{FP}\right)},\\ \mathrm{Precision}\;=\;\frac{\mathrm{TP}}{\left(\mathrm{TP}\;+\;\mathrm{FP}\right)},\\ \mathrm{Recall}\;=\;\frac{\mathrm{TN}}{\left(\mathrm{TN}\;+\;\mathrm{FN}\right)},\\ \text{F}1-\mathrm{Score}\;=\;\frac{2\;*\;\left(\mathrm{Precision}*\mathrm{Recall}\right)}{\left(\mathrm{Precision}\;+\;\mathrm{Recall}\right)}. \end{array}$$

All the experimentation is done using the freely accessible Google Colaboratory GPU environment. Each of the models is implemented using Python3 Keras library, with TensorFlow as backend. All the models are evaluated 10 times with 10 different random splits, where in each split 80% of the data is kept for training purpose (training data) and the rest for testing (testing data). The actual model training is done using 90% of the training data, with 10% of the training data kept as the validation set, which is used to perform early stopping, in order to avoid overfitting. The Convolution and Pooling parts of each of the model are followed by 3 fully connected layers (4096, 4096, and 1000), each with ReLU activation and finally a single-node prediction layer with Sigmoid activation function. The model optimization is performed using Stochastic Gradient Descent optimizer, with 0.001 learning rate and 0.9 momentum.

**Figure 4:**
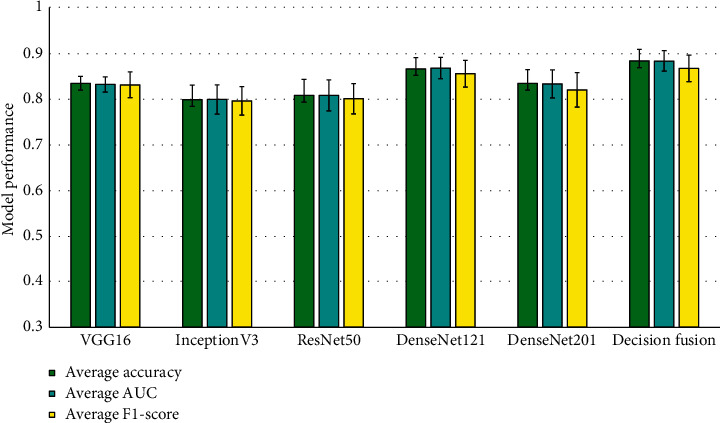
Average overall behavior of each individual model and the decision fusion model.

The overall behavior of the models can be observed by considering the Accuracy, AUC, and F1-Score of each of the models. In Figure 4, the average behavior of each of the models is shown, along with their corresponding 95% confidence intervals.

From Figure 4, it can be observed that the decision fusion model outperforms each of the individual models, achieving the highest average Accuracy, AUC, and F1-Score of 0.8834, 0.8832, and 0.867, respectively. Among the individual models, DenseNet121 performs the best.

**Figure 5:**
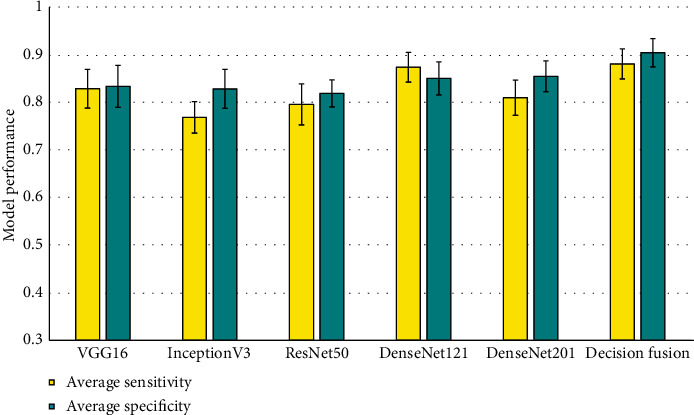
Average Sensitivity and Specificity of the Deep CNN based prediction models.

In case of average Sensitivity and Specificity also, the decision fusion based approach shows much better performance as compared to all the other individual models, achieving scores of 0.8813 and 0.9051, respectively, as shown in Figure 5. One important observation to be made here is that the average Specificity is improved greatly by the decision fusion based approach, suggesting a better False Positive Rate than any individual model.

**Figure 6:**
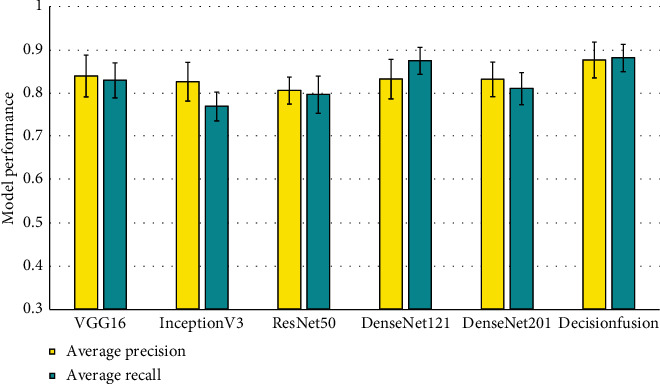
Average Precision and Recall of the Deep CNN based prediction models.

In case of average Precision and Recall also, similar trends can be observed, as shown in Figure 6. The decision fusion based approach outperforms each of the individual models. It can be observed from Figure 6 that the average Precision is shown to have good improvement over the individual models, which follows from the fact that the decision fusion model has a much better False Positive Rate. The training time and prediction time of one sample for each individual model, along with the proposed decision fusion model, are given in Table 1. It is important to note here that the training time is a one time cost, and in practice if such an approach is utilized, then only single sample prediction time will be of importance.

From the above experimental results, it is clear that Deep CNN based predictive models can be of very high value with regard to COVID19 identification from CT images. More specifically, the simple idea of decision fusion can improve the performance of Deep CNN models quite drastically, achieving above 86% results with respect to all the performance metrics under consideration. One more important observation is that the proposed approach has a very good reduction of False Positive Rate, which suggests its potential for use in real screening scenarios. One more important benefit of such a screening approach is that, apart from the time required to generate CT images (which is typically around 30 minutes), once the models are trained, prediction on each individual case can be performed in a matter of seconds, thereby drastically reducing the testing time for the patients.

## 4. Conclusion and Future Works

In this work, an experimental evaluation of existing Deep CNN based image classification approaches is presented in order to identify COVID19 positive cases from chest CT scan images. Moreover, a decision fusion based approach is also proposed, which combines the predictions of each of the individual Deep CNN models, in order to improve the predictive performance. From the extensive experimentations, it is observed that the proposed approach can achieve very impressive results, with above 86% in terms of every performance metric under consideration, while having a good reduction of the number of False Positives. From the experimental observations, it is clear that Deep CNN based approaches can potentially have a huge impact on the spread control of COVID19 by providing fast screening. With DL based approaches being used widely in other medical imaging tasks, it is high time for such approaches to be used in the screening process of the current pandemic as well.

In this work, only axial slices from CT images were used; however, it will be interesting to see how inclusion of other slices contributes to giving further information from the images. Also, with the availability of CT images with labeled information of other lung diseases, combining with COVID19 CT images might give more reliable systems. For now, we consider these are the limitations of the used dataset and these limitations will be addressed in the future work.

Furthermore, although the proposed approach shows great promise, there is still quite a bit of room for potentially improving the predictive performance of the approach. Recently, ideas like Transfer Learning, Image Augmentation, and Feature Level Fusion have been shown to boost the performance of DL based models drastically. These ideas are to be explored as part of the future work.

## Figures and Tables

**Table 1:** Training time and prediction time for the models.

Model	Training time (sec)	Prediction time for one sample (sec)
VGG16	2538.306655	0.0112183094
InceptionV3	3606.996002	0.02604055405
Resnet50	3338.539274	0.02051854134
DenseNet121	4490.50542	0.0279135704
DenseNet201	5721.441791	0.05062174797
Decision fusion	19807.89322	0.1363320236

## Data Availability

Publicly available COVID19 CT scan dataset has been used in this work (collected as of April 5, 2020), which is available at https://github.com/UCSD-AI4H/COVID-CT.
